# RNA Biogenesis Instructs Functional Inter-Chromosomal Genome Architecture

**DOI:** 10.3389/fgene.2021.645863

**Published:** 2021-03-01

**Authors:** Alessandro Bertero

**Affiliations:** Department of Laboratory Medicine and Pathology, Institute for Stem Cell and Regenerative Medicine, University of Washington, Seattle, WA, United States

**Keywords:** transcription, splicing, chromatin, genome organization, cardiomyocyte

## Abstract

Three-dimensional (3D) genome organization has emerged as an important layer of gene regulation in development and disease. The functional properties of chromatin folding within individual chromosomes (i.e., intra-chromosomal or in *cis*) have been studied extensively. On the other hand, interactions across different chromosomes (i.e., inter-chromosomal or in *trans*) have received less attention, being often regarded as background noise or technical artifacts. This viewpoint has been challenged by emerging evidence of functional relationships between specific *trans* chromatin interactions and epigenetic control, transcription, and splicing. Therefore, it is an intriguing possibility that the key processes involved in the biogenesis of RNAs may both shape and be in turn influenced by inter-chromosomal genome architecture. Here I present the rationale behind this hypothesis, and discuss a potential experimental framework aimed at its formal testing. I present a specific example in the cardiac myocyte, a well-studied post-mitotic cell whose development and response to stress are associated with marked rearrangements of chromatin topology both in *cis* and in *trans*. I argue that RNA polymerase II clusters (i.e., transcription factories) and foci of the cardiac-specific splicing regulator RBM20 (i.e., splicing factories) exemplify the existence of *trans*-interacting chromatin domains (TIDs) with important roles in cellular homeostasis. Overall, I propose that inter-molecular 3D proximity between co-regulated nucleic acids may be a pervasive functional mechanism in biology.

## Introduction

RNA biogenesis is a complex, multi-step process that largely takes place in the nucleus. Following transcription by RNA polymerases, most RNA species undergo extensive chemical modifications that are vital to their function. In eukaryotes, primary transcripts of messenger RNAs (pre-mRNAs) are 5'-capped, spliced, and finally 3'-polyadenylated and cleaved, altogether generating mature mRNAs. It is well established that these central steps of mRNA biogenesis occur in close proximity and at the same time and place as transcription (i.e., “co-transcriptionally”; reviewed in Perales and Bentley, 2009). Over a hundred additional RNA modifications are known (Boccaletto et al., 2018). At least some of these can be found on eukaryotic mRNAs, most notably for its abundance *N*^6^-methyladenosine (m^6^A), but also *N*^1^-methyladenosine (m^1^A), 5-methylcytosine (m^5^C), and pseudouridine (reviewed in Zhao et al., 2017). Emerging evidence suggests that many of these so-called “post-transcriptional” mRNA modifications actually take place largely co-transcriptionally. For instance, the dynamic writing and erasing of m^6^A is essentially complete before mRNAs are released from chromatin (Bartosovic et al., 2017; Ke et al., 2017a). This may be explained by recruitment of m^6^A “writers” by DNA polymerase II (Slobodin et al., 2017), epigenetic histone marks of transcriptional elongation (Huang et al., 2019), and/or specific transcription factors (Barbieri et al., 2017; Bertero et al., 2018). In turn, m^6^A deposition can affect alternative splicing and polyadenylation (Dominissini et al., 2012; Ke et al., 2015; Yue et al., 2018). Overall, mRNA biogenesis pathways are closely intertwined both spatially and functionally, and largely take place at the site of transcription.

The complexity of nuclear regulations of gene expression is staggering. Taking *Homo sapiens* as an example, there are at least 1,600 proteins likely functioning as transcription factors (Lambert et al., 2018), and over 250 splicing regulators (Jurica and Moore, 2003; Barbosa-Morais et al., 2006). Each of these molecules can affect the expression of tens to hundreds of genes with remarkable specificity despite having to locate their targets in a huge genomic and transcriptomic sampling space (~6.4 billion DNA nucleotides, up to 10% of which may be transcribed at a given time; Pertea, 2012), all the while being expressed at relatively modest copy numbers (estimated to be as low as a few thousand per cell in the case of transcription factors; Biggin, 2011). How this is all achieved in a time- and energy-effective manner remains a key open question.

An emerging topic in cell biology is that certain biochemical reactions are spatially organized in membraneless subcompartments formed by biomolecular condensates (reviewed in Banani et al., 2017). One of the first described and perhaps the best example of such structures is the nucleolus, the main site of ribosomal RNA (rRNA) biogenesis where most rDNA genes colocalize with highly concentrated factors implicated in rRNA transcription and processing (reviewed in Pederson, 2011). Many other conceptually similar nuclear structures have been described, including Cajal bodies, promyelocytic leukemia (PML) bodies, nuclear speckles, and histone locus bodies, some of which are still very poorly understood (reviewed in Mao et al., 2011).

With regards to mRNA biogenesis, over 20years ago Peter Cook and Ana Pombo proposed that transcription may be largely spatially restricted to “transcription factories” (Cook, 1995; Pombo et al., 2000). These correspond to concentrated foci of RNA polymerase II (Pol II) and various other transcriptional regulators that collectively support highly productive transcriptional initiation and elongation. It was readily recognized that transcription factories may have a strong effect on genome organization, driving the assembly of intra- and inter-chromosomal clusters involving active loci (Sexton et al., 2007; Cook, 2010). Here, I broaden this hypothesis arguing that not just transcription but also splicing and possibly other layers of RNA biogenesis may collectively shape chromatin architecture. Specifically, I propose the existence of RNA factories-nucleated “*trans*-interacting chromatin domains” (TIDs) involving loci on different chromosomes whose nucleic acids are co-regulated by the same set of protein factors. I further propose that the resulting architecture is not just practical (i.e., it facilitates and stabilizes chromatin folding) but also functional, by which I mean that it promotes the efficacy and fidelity of RNA biogenesis.

I begin by extensively reviewing the main features of inter-chromosomal genome architecture. I follow this with a shorter discussion of the possible role of genetic variants in *trans*, and with some specific examples borrowed from cardiomyocyte biology. Having outlined the supporting evidence, I then flesh out my hypothesis and propose an experimental framework for its formal testing. I conclude by discussing open questions in the area. Throughout I focus on mammalian genome regulation and architecture since it is well established that it involves unique mechanisms not seen in other model organisms such as invertebrates, plants, and yeasts (which in turn have their own peculiarities). Nevertheless, I present selected examples from the broader spectrum of eukaryotic life, as I speculate that the general principle behind the notion of TIDs may be broadly applicable.

## Inter-Chromosomal Genome Architecture

Over the last decade, technological advancements in the field of chromatin architecture have firmly established that mammalian interphase chromosomes are not randomly dispersed in the nucleus, but rather highly organized (reviewed in Rowley and Corces, 2018; Kempfer and Pombo, 2020). Most of our current understanding of genome topology is focused on intra-chromosomal (*cis*) interactions resulting from a variety of hierarchical features at different genomic scales. These include various kinds of DNA loops (such as promoter-enhancer pairing), topologically-associating domains (TADs; sub-megabase regions of preferential self-interaction), and A/B compartmentalization (chromosome-wide separation of active/inactive chromatin due to inter-TAD contacts). A large body of work has recently emerged to show that while a large fraction of intra-chromosomal genome structure is largely invariant across cell types and states, dynamic changes do occur at all levels and can contribute to gene regulation both during development and in disease (reviewed in Krumm and Duan, 2018; Zheng and Xie, 2019).

In contrast to this, inter-chromosomal (*trans*) interactions are much less understood and studied. The motivations are at least three-fold. First, there is a widespread belief in the community that most inter-chromosomal interactions are just noise resulting from Brownian movements of chromatin, and/or technical artifacts arising from proximity ligation-based methods (which dominate the field of chromatin conformation capture, 3C). Indeed, one commonly used quality control measure for 3C-type studies is the ratio of *cis/trans* interactions, which is expected to be high. While *trans* interactions are certainly more noisy than *cis* ones, as discussed below, abundant evidence indicates that there is nevertheless signal worth measuring. Which brings us to the second problem, namely the relative immaturity of statistical and computational approaches to confidently identify *trans* interactions. Indeed, due to their different biophysical nature *trans* interactions cannot be simply treated analytically with the now well-established methods developed for *cis* ones (which for instance rely on normalizing contact counts based on the linear genomic distance to account for the varying probability of random interactions). Finally, compounding on this limitation is the fact that *trans* interactions are less frequent than *cis* ones by a factor of ~2–5 (depending on the cell type and assay), while being also spread across a much wider contact matrix 2D space (and even a broader one if we consider three-way interactions). This results in data that is very sparse unless a prohibitively expensive sequencing depth is utilized, particularly in the case of genome-wide studies. Statistical approaches to detect *trans* interactions have therefore either low power (few data points) or low resolution (genomic bins of large size are used to aggregate the sparse interaction counts). As a result of these three main factors, *trans* interactions are often dismissed early during the analysis or only used for very basic broad assessments of chromosome territories.

That all being said, some key advances have already been made in our understanding of inter-chromosomal genome architecture. These are summarized in the subsections that follow.

### Chromosome Territories

Mammalian interphase chromosomes are not fully intermixed yet occupy mostly distinct nuclear domains referred to as “chromosome territories” (reviewed in Cremer and Cremer, 2010). The territorialization of chromatin was hypothesized in the late 19th and early 20th century by Carl Rabl and Theodor Boveri, and experimentally demonstrated through microscopical observations by Thomas and Christoph Cremer in the 1980s (Cremer et al., 1982). Some 30years later, genome-wide 3C analysis (Hi-C) by Lieberman-Aiden et al. (2009) showed that the intra-chromosomal contact probability is much greater than the average contact probability between different chromosomes, consistently with the existence of chromosome territories. Chromosome territories have been observed in multiple species including yeasts (Duan et al., 2010) and plants (Dong et al., 2018), indicating that this is perhaps a universal feature of nuclear organization in multicellular organisms. Chromosome territories are not randomly positioned in the nucleus yet present preferential relative placement (Croft et al., 1999; Nagele et al., 1999; Cremer et al., 2001), a property that may partially explain the reproducible outcome of certain common chromosomal translocations (Roix et al., 2003). The distribution of chromosome territories is also somewhat tissue-specific (Parada et al., 2004; Bolzer et al., 2005), and is transmitted through mitosis (Gerlich et al., 2003). Chromosome territory dynamics may thus influence (or be influenced by) cell identity, and contribute to the epigenetic memory stabilizing a given state.

The well-established concept of chromosome territories is perhaps the motivation leading many to dismiss inter-chromosomal architecture, relegating its possible relevance to “exotic” examples such as sperm cells (which are characterized by a high frequency of both extra-long-range *cis* interactions and *trans* contacts; Ke et al., 2017b) or to non-mammalian cells (such as the KNOT structure in *Arabidopsis thaliana*, an established heat-shock responsive inter-chromosomal structure; Dong et al., 2018; Sun et al., 2020). The general skepticism about early reports of *trans* interactions frequency in Hi-C experiments as high as 60–70% was well funded: it was later shown that in-solution proximity ligation leads to many spurious ligation events, a problem that can be bypassed for instance by performing in-nucleus proximity ligation (Nagano et al., 2015). Nevertheless, even using this refined protocol the frequency of *trans* interactions remained consistently between 10 and 15% for both human and mice samples (Nagano et al., 2015). An even more rigorous assessment of multi-way chromosomal conformation through chromosomal walks (C-walks) confirmed a frequency of 7–10% inter-chromosomal interactions (Olivares-Chauvet et al., 2016). Even assuming that this is still an overestimation of *bona fide* interactions that may in truth be as limited as to ~5% of the total (and as discussed below this fraction seems to be much larger in certain cell types), this would still account for 1/20th of the data. Is it not worth to analyze it beyond just confirming the well-established concept of chromosome territories?

### Nuclear Subcompartments

When the question posed above has been answered in the affirmative, important insights have been made. Lieberman-Aiden et al. (2009) leveraged their aforementioned Hi-C data to show that small, gene-rich chromosomes and large, gene-poor chromosomes form distinct clusters that preferentially interact with each other. These findings confirmed earlier analyses by DNA fluorescent *in situ* hybridization (FISH; Boyle et al., 2001; Tanabe et al., 2002). This was an important hint that *trans* interactions happening at the borders of chromosome territories are not simply random, but may for instance relate to transcriptional activity.

Building upon this concept, Rao et al. (2014) found that incorporating *trans* interactions when determining chromatin compartmentalization features allowed to go beyond the simple A/B paradigm by identifying at least six subcompartments, each bearing a distinctive pattern of epigenetic features. While the dataset in question was exceptional for its sequencing depth (4.9 billion contacts), this same feat can now be achieved from “conventionally-sized” datasets with moderate coverage using the SNIPER algorithm (Xiong and Ma, 2019). This computational approach leverages once again on inter-chromosomal interactions, imputing them based on a statistical model when the data is too sparse, and identifies subcompartments with defined epigenetic characteristics and, in some cases, strong cell specificity. This is another strong piece of evidence indicating that *trans* interactions correlate with gene expression regulation dynamics.

Kaufmann et al. (2015) performed a comprehensive analysis of inter-chromosomal interaction networks comparing Hi-C data for mouse and human embryonic stem cells (mESCs and hESCs). Remarkably, this pointed at ~70 and ~40% of the genome as being involved in at least one inter-chromosomal interaction in mESCs and hESCs, respectively, corresponding to ~30% of genes in both species. Such contacts are not homogeneously distributed: similarly to what previously observed for *Saccharomyces cerevisiae* (Kruse et al., 2013), the authors found a high degree of clustering within the interaction networks. In hESCs, small, gene dense chromosomes dominate the network, interacting predominantly with themselves in agreement with the aforementioned findings of Lieberman-Aiden et al. (2009). Additionally, both mouse and human chromosomes interact more strongly at centromeres. Domains involved in human intra-chromosomal interactions (but not mouse ones) are enriched in active histone marks, suggesting their involvement with human-specific transcriptional dynamics. Further supporting this notion, inter-chromosomal interactions between human gene pairs significantly correlate with similarity in both function and co-expression. In this context, approximately half of all human *trans*-interacting genes are bound by the CTCF/cohesin complex, an established master regulator of both intra- and inter-chromosomal interactions (reviewed in Ong and Corces, 2014). Remarkably, human and mouse inter-chromosomal network seem to be very poorly conserved (i.e., not more significantly that would be expected at random), in sharp contrast with the strong conservation of intra-chromosomal architecture between these two species (Dixon et al., 2012). It is therefore tempting to speculate that rearrangements in inter-chromosomal architecture may have played a role in human-specific evolutionary changes, perhaps by altering gene co-regulation through novel nuclear “hubs.”

Aiming to increase the signal to noise ratio of Hi-C measurements, particularly in the context of inter-chromosomal interactions, Kalhor et al. (2012) developed tethered conformation capture (TCC), which prevents Brownian interactions between crosslinked DNA complexes during proximity ligation. This approach resulted in a proportion of inter-chromosomal interactions of 25–30% of the total contacts, an approximately two-fold reduction compared to non-tethered, in solution Hi-C. To bypass the inherent inability of bulk Hi-C to detect multivalent chromatin interactions happening in individual cells, the authors also developed a modeling approach that constructs a population of three-dimensional (3D) genome structures that collectively best explain the observed interactions. Analyses of *trans* contacts revealed three major aspects: (1) each human chromosome possesses a few regions collectively explaining the majority of *trans* interactions; (2) such regions are characterized by high transcriptional activity; and (3) they interact with each other in a largely indiscriminate fashion. This led the authors to speculate that such domains intermingle chiefly because they happen to all be accessible within the nuclear interior, “escaping” the constraints of their respective chromosome compartments. However, refinements in the quantitative analyses of 3D structure population models showed that some inter-chromosomal interactions are actually relatively specific and liked to gene co-regulation (Dai et al., 2016). Indeed, the authors identified nearly four thousand “regulatory communities”: clusters of chromatin domains enriched for specific regulatory factors. These include Pol II and transcription factors, RNA polymerase III (Pol III), and the polycomb binding protein YY1, suggesting that the relevant regulatory communities may represent transcription factories, transfer RNA factories, and polycomb domains, respectively. Notably, ~80% of regulatory communities contain domains from multiple chromosomes, indicating an extensive degree of inter-chromosomal architecture.

Proximity ligation-based approaches to map genome organization are designed to capture close proximity between DNA fragments. Therefore, they are relatively blind to intra- and inter-chromosomal architecture revolving around relatively large nuclear bodies, which can range from ~0.5 to ~2μm. To bypass this limitation, Quinodoz et al. (2018) developed a ligation-independent approach called split-pool recognition of interactions by tag extension (SPRITE), which allows the identification of very large chromatin clusters with even more than a thousand DNA fragments. By applying this approach to human cells, they revealed how a large fraction of inter-chromosomal interactions revolve around two main hubs: a “nucleolar hub” enriched for peri-centrosomal, gene-poor domains stably interacting with rDNA genes and rRNA transcripts, and a “speckle hub” partial to highly transcribed, gene-rich regions dynamically coming into contact with spliceosomal RNAs and other mRNA processing factors (all characteristics of so-called “nuclear speckles”; Galganski et al., 2017). This elegant study extended earlier observation that actively transcribed, gene-dense regions can loop out from the center of chromosome territories (Mahy et al., 2002; Branco and Pombo, 2006), indicating that at least some of these converge onto nuclear speckles. Notably, similar results have been obtained with other ligation-free methods such as genome architecture mapping (GAM; Beagrie et al., 2017) and tyramide signal amplification sequencing (TSA-seq; Chen et al., 2018).

Interestingly, a subsequent study showed that inter-chromosomal interactions mapped by both TCC and SPRITE have a strong GC sequence bias (Jabbari et al., 2019), in agreement with earlier observations on Hi-C data (Yaffe and Tanay, 2011). Therefore, it is possible that aggregation into nuclear bodies may favor flexible, nucleosome poor, and loosely packed genomic regions, all characteristics of GC-rich domains, while rigid and compacted AT-rich regions are instead enriched at heterochromatic sites at the nuclear lamina (Jabbari and Bernardi, 2017).

**Figure 1:**
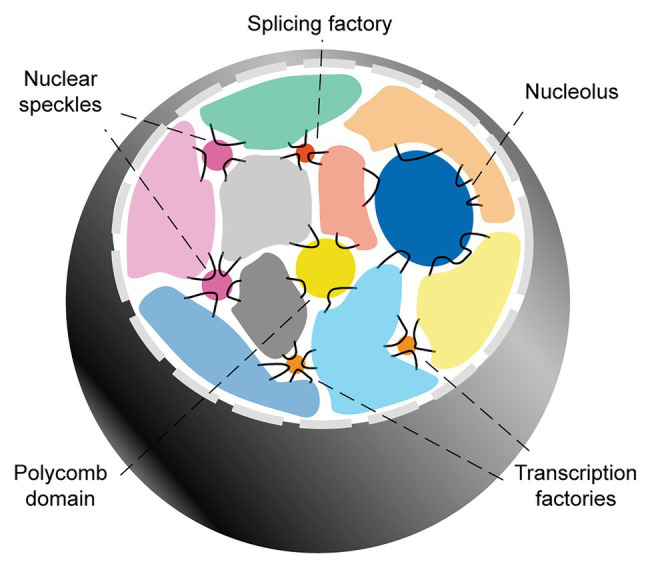
Inter-chromosomal genome organization. Interphase mammalian nuclei are characterized by chromosome territories. Inter-chromosomal interactions mainly involve genomic domains that extrude into the inter-chromosomal space and engage with a variety of membranelles structures involved in gene regulation and RNA biogenesis.

The overall picture emerging from these seminal studies is that while chromosome territories greatly limit the possibility for inter-chromosomal interactions, they do not present hard boundaries. Among the regions able to overcome chromosome territories-mediated topological restrictions, several genomic domains (particularly flexible, GC-rich regions characterized by high transcriptional activity), can extrude to the surface and engage with each other in the proximity of transcription factories, tRNA factories, polycomb domains, the nucleolus, nuclear speckles, and/or other yet-to-be clarified nuclear subcompartments (Figure 1).

In the following subsections, I provide a more in-depth review of the nuclear subcompartments that are most relevant to the hypothesis of this article. For this, I focus on structures involved in the biogenesis of mRNAs. The relationship between chromatin organization and other nuclear subcompartments involved in non-coding RNA biogenesis, such as the nucleolus and tRNA factories, is reviewed extensively elsewhere (Pederson, 2011; Kirkland et al., 2013; Bersaglieri and Santoro, 2019).

#### Transcription Factories

As just discussed, highly transcribed genomic domains are those most often involved in inter-chromosomal interactions. Accordingly, inhibition of transcription reduces chromosome intermingling (Branco and Pombo, 2006). Moreover, mapping of Pol II-mediated chromatin interactions showed that these are not just intra- but also inter-chromosomal (Li et al., 2012). *Trans* interactions between active genes may be indiscriminate and dictated by the random motion of open chromatin in the euchromatic nuclear interior, or could be highly specific. A few examples of the latter category have accumulated over the years, indicating that both mechanisms likely contribute to inter-chromosomal architecture.

Following early indirect evidence and the formulation of the “transcription factory” hypothesis by Cook (1995) and Pombo et al. (2000), the laboratory of Peter Fraser was the first to formally show that transcribed genes in *cis*- and in *trans*- often colocalize at the site of transcription factories (Osborne et al., 2004). Through RNA FISH analyses the frequency of this colocalization was estimated to be 40–60% for several intra-chromosomal interactions involving the *Hbb* (beta-globin) gene, and 7% for the inter-chromosomal interaction between *Hbb* and *Hba* (alpha-globin), which is substantially higher than the expected frequency for a random interaction (~1%). In this context *cis* interactions involving *Hbb* require initiation of transcription, arguing for an active recruitment model (Mitchell and Fraser, 2008). Subsequent genome-wide studies using 3C-based approaches validated the various *cis* interactions of the *Hbb* locus (Simonis et al., 2006), and found a strong proportion of *trans* interactions for *Hbb* in the fetal liver (~40% of all contacts, a rate ~10% higher than what is seen in non-*Hbb*-expressing brain tissue; Pink et al., 2010). Enhanced chromatin immunoprecipitation followed by 3C-on-chip (e4C) indicated that a striking ~90% of all interactions involving Pol II-enriched *Hbb* or *Hba* involve genes on other chromosomes (Schoenfelder et al., 2010). While these *trans*-interacting gene networks are for the most part distinct, some genes were shown to significantly interact with both *Hbb* and *Hba* at the same transcription factory (*Slca1*, *Kel*, and *Tfrc*). Remarkably, both the recruitment of *Hbb* to transcription factories and its colocalization with its *trans* partners *Hba* and *Epb4.9* requires the erythroid transcription factor Klf1 (Schoenfelder et al., 2010). Klf1 is enriched in ~40 foci per nucleus, corresponding to only ~10–20% of transcription factories. These findings strongly argued for the existence of specialized transcription factories “bookmarked” by high levels of Klf1.

Compelling evidence for another specialized transcription factory came once again from the Fraser lab, which showed that induction of immediate early genes in B cells leads to frequent repositioning of the proto-oncogene *Myc* to the same transcription factory occupied by *Igh*, a well-known inter-chromosomal translocation partner (Osborne et al., 2007). Such association was reported at a frequency of ~25%, which is similar to that of the intra-chromosomal colocalization of *Igh* with *Fos*, and up to 10-fold higher than that for *Igh* and other genes on the same chromosome as *Myc*, overall suggesting a strong specificity. Other notable inter-chromosomal gene-gene interactions in either putative or *bona fide* transcription factories include the Oct4 and Nanog-dependent interactions between the *Nanog* gene itself and several other pluripotency factors in mESCs (de Wit et al., 2013), and the Brg1- and Stat3-dependent clustering of *Gfap* and other co-regulated genes such as *Osmr* in differentiating astrocytes (Takizawa et al., 2008; Ito et al., 2016, 2018).

**Figure 2:**
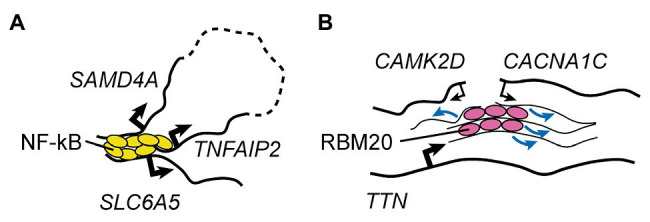
RNA Factories. **(A)** A representative, well-understood transcription factory involved in the control of TNFα-responsive genes in endothelial cells (Papantonis et al., 2010, 2012; Fanucchi et al., 2013). Rapidly after TNFα treatment, binding of NF-κB leads to clustering of two genes located on the same chromosome but separated by ~50Mb (dashed line), and of a third gene found on a different chromosome. This mechanism is key to transcriptional activation of all three factors in the same cell. **(B)** The muscle-specific RBM20 splicing factory (Bertero et al., 2019a). Binding of RBM20 to the many sites on its core target, the *TTN* pre-mRNA, nucleates foci that lead to clustering of other targets, promoting the regulation of their splicing. Black arrows indicate transcriptional activity (thin lines: weak; thick lines: strong), and blue arrows indicate regulation of splicing.

One final but particularly remarkable example of specialized transcription factory is the one involving TNFα-responsive genes in endothelial cells (Figure 2A; Papantonis et al., 2010, 2012). It was shown that 10min after stimulation with TNFα the promoters of two genes located ~50mb apart on the same chromosome (*SAMD4A* and *TNFAIP2*) and a third gene on a different chromosome (*SLC6A5*) can associate as part of a NF-κB-dependent multigene cluster (Papantonis et al., 2010, 2012). This happens in ~5% of the cells, indicating that it is a relatively reproducible event (Fanucchi et al., 2013). Using this model system, Fanucchi et al. (2013) set out to test whether the formation of these intra- and inter-chromosomal contacts are required for cotranscription of the interacting genes. By perturbing, in turn, each of the three chromatin interaction sites through cleavage of the relevant genomic DNA, they uncovered important *trans*-acting effects on NF-κB-dependent gene expression. Indeed, cleavage of a single allele of *SAMD4A* resulted in virtually no transcription of both *TNFAIP2* and *SLC6A5* after TNFα stimulation. Importantly, this was observed in cells where the remaining copy of *SAMD4A* was sufficient to maintain wild type-like levels of SAMD4A protein throughout the duration of the experiment, excluding the possibility that this factor is required for the transcription of *TNFAIP2* and *SLC6A5*. The authors further observed that not all genes in the cluster were equally important for each other’s transcription: cleavage of *TNFAIP2* only reduced *SLC6A5* expression, while cleavage of *SLC6A5* did not affect the other two genes. Overall, the authors proposed a hierarchical model whereby the dominant member of the NF-κB multigene complex, *SAMD4A*, organizes transcription of subordinate genes through the establishment of both long-range intra-chromosomal interactions and inter-chromosomal contacts. To my knowledge, this is the only mechanistic study to date that demonstrated how a specialized transcriptional factory can be more than just a structural feature of the nucleus, representing a key functional entity that promotes gene co-regulation.

RNA factories may not just characterize interphase chromosomes. Peter Cook first theorized that transcription factories from homologue chromosomes play a key role in their pairing during mitosis (Cook, 1997). In this context, the formation of inter-chromosomal interactions between loci sharing the same set of protein regulators would stabilize the association of partially-condensed homologue chromosomes. Fulfilling this prophecy, recent work demonstrated that homologue chromosome pairing in yeast relies on ribonucleoprotein (RNP) complexes involving locally-transcribed, meiosis-specific long non-coding RNAs (lncRNAs; Ding et al., 2019).

Besides these examples in mammalian systems, inter-chromosomal interactions involving co-regulated genes have been observed in organism as different as yeasts (Homouz and Kudlicki, 2013), wheat (Concia et al., 2020), and sea urchins (Matsushita et al., 2017). This suggests that the formation of transcription factories that reproducibly involve the same loci (henceforward referred to as “zip coded transcription factories”) is either an evolutionarily conserved mechanism or an emergent property of gene regulation across genomes of different complexity and size. However, the pervasiveness of this mechanism remains to be established.

#### Nuclear Speckles

Inter-chromosomal interactions between co-regulated active genes are not limited to those occurring at transcription factories. As discussed, nuclear speckles (which are functionally very distinct from Pol II clusters; Galganski et al., 2017), represent another key subcompartment enriched for *trans* interactions (Quinodoz et al., 2018). Nevertheless, only a handful of specific cases have been studied in detail to date.

One such example is the estrogen receptor α-induced interaction of *TFF1* and *GREB1* in mammary epithelial cells (Hu et al., 2008). This involves a two-step process relying first on actin/myosin1/DLC1-mediated cytoskeletal dynamics to reorganize chromosome territories, and then on the histone lysine demethylase LSD1 to induce the *trans* association of *TFF1* and *GREB1* in the context of nuclear speckles (Hu et al., 2008). Another example is the aforementioned interaction of alpha- and beta-globin genes with each other as well as with other erythrocyte genes. Challenging the notion that these often share a transcription factory, Brown et al. (2006, 2008) found that a large fraction of these inter-chromosomal associations happen around nuclear speckles.

Whether colocalization at the same nuclear speckle for co-regulated genes promotes transcriptional and/or post-transcriptional regulation has not, to my knowledge, been yet formally tested.

#### Splicing Factories

While much less established, the role of mRNA biogenesis steps besides transcription in inter-chromosomal regulation is beginning to emerge. Indeed, my colleagues and I recently showed that in cardiomyocytes derived from hESCs (hESC-CMs) foci of the muscle-specific splicing factor RBM20 mediate inter-chromosomal interactions between some of its key target loci (Figure 2B; Bertero et al., 2019a). We also mechanistically dissected the process by showing that RBM20 foci form only when scaffolded by its primary target, the pre-mRNA of the giant sarcomeric factor *TTN*, which is known to contain more than 100 RBM20 binding sites (Maatz et al., 2014). In cells lacking the *TTN* pre-mRNA due to a promoter deletion, and therefore with no RBM20 foci, the *TTN* gene no longer engages in *trans* interactions with the RBM20 target genes *CAMK2D* and *CACNA1C*. While in these conditions the expression and nuclear localization of RBM20 are unchanged, the RBM20-dependent alternative splicing events on both *CAMK2D* and *CACNA1C* is markedly impaired. Importantly, deletion of the titin protein product but not of the *TTN* pre-mRNA (through the insertion of a premature stop codon that does not lead to nonsense-mediated decay) had no effect on the formation of RBM20 foci and on the splicing of *CAMK2D* and *CACNA1C.* This excludes the possibility that titin is somehow involved in the control of RBM20 localization and/or activity, and indicates that the *TTN* pre-mRNA has specific *trans*-acting effects.

Overall, we concluded that RBM20 foci represent the first example, to the best of our knowledge, of a nuclear subcompartment specialized in the regulation of splicing for a defined subset of genes located across multiple chromosomes, in other words, a *trans*-acting “zip coded splicing factory.” Whether this is an exceptional case or the first of many examples of this type of regulation is yet to be determined.

#### *Trans*-Acting Regulatory Regions

On top of engagement of co-regulated genes in transcription and/or RNA processing factories, there are several instances of specific regulatory *trans* interactions involving a single gene and one or more regulatory sequences that either repress or promote its expression. Repressive regulations include the inter-chromosomal interaction between the T helper cell 2 locus control region (TH2 LCR) and the *IFN-γ* gene (Spilianakis et al., 2005). This interaction is very strong in naïve CD4 positive T cells, and is relieved after specification of either TH2 or TH1 cells, the latter leading to a novel intra-chromosomal interaction of *IFN-γ* that promotes its expression. Thus, the TH2 LCR is believed to create a repressive-yet-poised chromatin hub key to the rapid activation of one of the two T helper specification programs. Initiation of mesendoderm differentiation is similarly regulated by an Oct4-dependent switch of the *Sox17* enhancer from a locked inter-chromosomal conformation (involving the *Sox2* gene) to an active intra-chromosomal engagement with *Sox17* (Abboud et al., 2015). Myogenesis is also temporally regulated by the inter-chromosomal sequestration of key regulatory regions for late muscle genes, which relies on the master transcription factor MyoD1 and is required to prevent their premature activation (Harada et al., 2015). Another interesting example is the interaction between the well-studied Igf2/H19 imprinting control region (ICR) and over 100 sequences from all autosomes, leading to epigenetic regulation in *trans* (Zhao et al., 2006). One such interaction involving the maternal ICR and the mouse Wsb1/Nsf1 Locus relies on CTCF for its stabilization, and represses Wsb1/Nsf1 expression (Ling et al., 2006). In porcine cells, the IGF2/H19 ICR is found in the proximity of other imprinted genes such as *DLK1* and *MEG3* (Lahbib-Mansais et al., 2016), in agreement with the strong overrepresentation of imprinted domains in the genome-wide interactions involving this ICR in the mouse (Zhao et al., 2006).

Besides these repressive *trans* chromatin structures, there are multiple examples of inter-chromosomal regulations that promote gene expression. Activation of the *IFN-β* gene relies on *trans* interactions with up to three genomic regions that bring limiting amounts of the key viral-induced transcription factor NF-κB onto the *IFN-β* enhancer, allowing the formation of an enhanceosome (Apostolou and Thanos, 2008). Similar inter-chromosomal enhancers promote expression of *Pax5* specifically in B cells (Fujita et al., 2017), and of *Tead4* in trophoblast stem cells (Tomikawa et al., 2020). One final example is perhaps the most extraordinary *trans* chromatin regulation described to date, namely the formation of the mouse olfactory receptor multi-chromosomal super-enhancer (Lomvardas et al., 2006; Markenscoff-Papadimitriou et al., 2014; Monahan et al., 2017). This structure, which includes 63 enhancers from 18 chromosomes (named Greek islands), is key to remove heterochromatin marks from a single olfactory receptor chosen stochastically among more than 1,000 such genes dispersed in various heterochromatin clusters. Formation and maintenance of this remarkable inter-chromosomal enhancer cluster requires the active involvement of the Greek islands-bound transcription factor Lhx2 and of its adaptor protein Ldb1 (Monahan et al., 2019).

Overall, it is now clear that inter-chromosomal chromatin architecture is not just restricted to active genes in more or less defined nuclear subcompartments or neighborhoods, but can extend to include complex multifactorial regulatory interactions generally mediated and stabilized by specific protein co-factors.

#### Polycomb Domains

A specific subtype of *trans*-interacting regulatory structure is presented by regions brought in proximity by the Polycomb protein complex (PcG), an important regulator of gene silencing through the deposition of the repressive histone mark histone 3 lysine 27 trimethylation (H3K27me3). PcG factors such as the catalytic subunit EZH2 are involved in both intra- and inter-chromosomal interactions (Tiwari et al., 2008). For instance, in mESCs primed for differentiation the homeobox gene clusters (Hox; key developmental regulators of antero-posterior patterning) become bivalently marked by H3K4me3 and H3K27me3, corresponding to the onset of specific long-range intra- and inter-chromosomal interactions (Joshi et al., 2015). Notably, these Hox “polycomb domains” are localized away from the nuclear lamina and within the A compartment (Vieux-Rochas et al., 2015), suggesting that the formation of a specific bivalently-marked chromatin sub-microenvironment within active chromatin may be key to maintain selective gene silencing while allowing for rapid activation of Hox factors upon developmental cues.

## *Trans*-Acting Expression Quantitative Trait Loci

Genetic variants associated with disease phenotypes are overwhelmingly non-coding, with <5% of trait-related single-nucleotide polymorphisms (SNPs) identified by genome-wide association studies (GWAS) representing non-synonymous substitutions in protein-coding genes (Eicher et al., 2015). It is generally thought that a large fraction of disease-associated non-coding variants may be explained by alterations in inter-chromosomal regulations such as promoter-enhancer interactions (Dekker and Mirny, 2016; Delaneau et al., 2019), possibly due to altered binding of transcription factors (Wong et al., 2017). Indeed, GWAS SNPs are very often associated with the expression levels of nearby genes, referred to as *cis* expression quantitative trait loci (*cis* eQTLs; Michaelson et al., 2009). However, eQTL analyses have also revealed a growing number of genetic variants that affect gene targets (eGenes) either far away on the same chromosome (separated by at 1–5Mb, depending on the study definition) or located on a different chromosome altogether (Heinig et al., 2010; Fehrmann et al., 2011; Innocenti et al., 2011; Fairfax et al., 2012; Grundberg et al., 2012; Liang et al., 2013; Battle and Montgomery, 2014; Kirsten et al., 2015). Such variants are therefore referred to as *trans* eQTL. These are difficult to identify with statistical confidence both because of the large multiple testing burden involved and due to a generally weaker effect on gene expression compared to *cis* eQTLs (McKenzie et al., 2014). Nevertheless, an eQTL meta-analysis including 5,311 patients was able to identify 1,513 significant *trans* eQTLs involving 346 SNPs and 430 genes (Westra et al., 2013). An even larger analysis of a single cohort of 6,111 individuals found 5,749 lead *trans* eQTLs affecting 4,958 genes (Joehanes et al., 2017).

**Figure 3:**
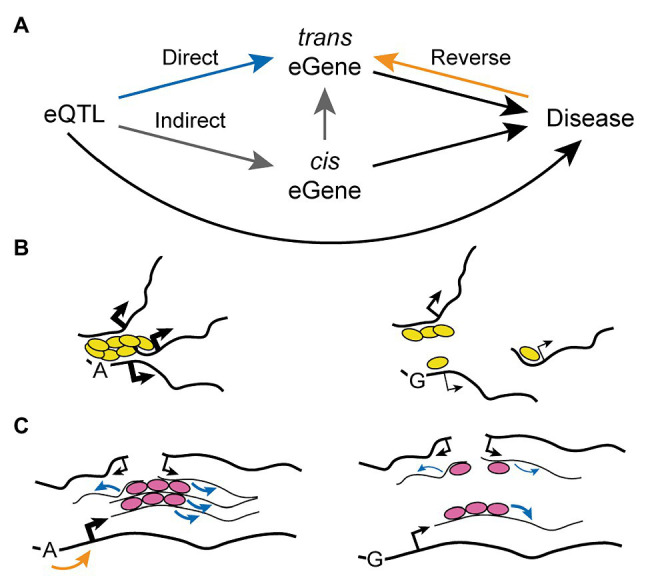
Mechanism of Action of *Trans*-Acting Expression Quantitative Loci. **(A)** The connection between a *trans* eQTL and its *trans* eGene can be mediated by three possibilities: direct regulation (blue path), indirect nuclear regulation through an intermediate *cis* eGene (gray path), or reverse regulation from a diseased state (black plus orange pathways). **(B,C)** Indirect regulation of *trans* eGenes by *trans* eQTL may in some cases be mediated by disruption of transcription factories **(B)** or splicing factories **(C)**. An allelic variant may decrease binding of *trans*-acting regulators (i.e., transcription factors, yellow ovals, or splicing factors, pink ovals) to core nucleic acids, disrupting their clustering and the resulting formation of *trans*-interacting chromatin domains and RNA factories (see Figure 4A). Black and blue arrows indicate transcriptional and splicing activity, respectively (thin lines: weak; thick lines: strong). The orange arrow indicates regulation of transcriptional initiation.

The mechanism of action of *trans* eQTLs remains unclear, but three major possibilities have been proposed so far (Figure 3A). First, the gene variant may directly affect the expression of the distal gene (*trans* eGene), perhaps as a result of an inter-chromosomal regulatory interaction such as those described in the above section “*Trans*-Acting Regulatory Regions”. Secondly, the gene variant may act indirectly through a third actor: a proximal gene (*cis* eGene) whose expression is directly regulated by the SNP and that in turn can directly modulate expression of the *trans* eGene. Proposed mechanisms behind the mediating action of such a *cis* eGene include its function as a transcription factor, micro RNA, or another regulatory non-coding RNA (Joehanes et al., 2017). Finally, the gene variant may be regulating the *trans* eGene even more indirectly as a result of “reverse causality”: a feedback mechanism arising from a diseased state caused directly by the SNP (for instance in the case of non-synonymous substitutions) or through modulation of a *cis* eGene. Indirect regulation or reverse causality can be tested through mediation analysis, which assesses whether a third factor (either a *cis* eGene or a phenotype) significantly accounts for the observed *trans* eQTL-*trans* eGene relationship. Using this approach it was found that 20–35% of *trans* eQTLs can be explained by the alteration of a mediating *cis* eGene (Pierce et al., 2014; Yao et al., 2017). Notably, over 80% of *trans* eQTLs also have an associated *cis* eGene (Yao et al., 2017), indicating that the proportion of “indirect *trans* eQTLs” may be even larger than what could be assessed with confidence by mediation analysis. The comprehensive genotype-tissue expression (GTEx) consortium reported similar observations, as 31.6% of lead *trans* eQTL identified across 27 tissues proved to be also *cis* eQTL, and 77% of these were predicted to contribute to the *trans* effect by mediation analysis (Aguet et al., 2020). Interestingly, Yao et al. (2017) found no evidence for the possibility of reverse causality as an underlying mechanism explaining *trans* eQTLs, suggesting that if this mechanism does contribute it is not a common and/or a strong factor.

In the study of Yao et al. (2017) some *trans* eQTLs were found to significantly regulate numerous genes and were thus called “*trans* eQTL hotspots.” These were also notable because the resulting gene regulation had a clear directional bias, with 65% of *trans* eGenes for a given *trans* eQTL hotspot being all up- or-down-regulated. This may be explained by the indirect alteration of a *cis* eGene with function of transcription factor activating or repressing a gene network containing the *trans* eGenes. For instance, this kind of mechanism was proposed for a *trans* eQTL which controls the expression of IFN-α-responsive genes through indirect alteration of the transcription factor *IKZF1* (Westra et al., 2013). However, Yao et al. (2017) found no significant enrichment for transcription factors in the *cis* eGenes linked to *trans* eQTL hotspots (only 2 out of 37). On the other hand, the authors found that approximately one third (13/37) of these *cis* eGenes shared the same regulatory motif with the *trans* eGenes. A similar enrichment of transcription factor motifs in the promoter of *trans* eGenes linked to *trans* eQTL has also been reported elsewhere (Joehanes et al., 2017). Thus, it is an intriguing possibility that a substantial fraction of *trans* eQTLs may act through an alternative mechanism that does not involve the protein product of a *cis* eGene, but rather shared regulatory mechanisms at the level of mRNA biogenesis. For instance, disruption of RNA factories due to impaired binding of *trans*-regulatory factors and/or to reduced abundance of core nucleic acids may impair *trans*-acting transcriptional and/or splicing defects (Figures 3B,C; refer to the model discussed in the below section “Mechanisms Leading to the Formation of RNA Factories”). Of note, a similar mechanism could in principle be involved in the action of *trans* eQTLs involving *cis* eGenes located far away on the same chromosome.

A recent study found evidence that human inter-individual correlation of chromatin regulatory activity driven by genetic variation occurs both intra- and inter-chromosomally, leading to the formation of *trans*-regulatory hubs, TRHs (Delaneau et al., 2019). The authors found several lines of evidence suggesting a link between this kind of regulation and inter-chromosomal genome architecture: (1) correlation in chromatin activity is enriched for genomic domains involved in inter-chromosomal interactions; (2) TRHs belong to the A or B compartment; (3) *trans* allelic correlation was observed in TRHs; and (4) some TRHs mediate *trans* eQTL effects. Along these lines, *trans* eQTLs identified by the GTEx consortium are specifically enriched for CTCF binding sites (Aguet et al., 2020). Overall, these findings add further evidence to the possibility that genetic variation may influence inter-chromosomal genome architecture through modulation of RNA biogenesis in *trans*.

## The Cardiomyocyte Nucleus: A Case in Point

While in the previous sections I introduced evidence for the interplay between RNA biogenesis and inter-chromosomal genome organization across many different biological systems, here I provide a cohesive case in point for a specific model: the mammalian cardiac myocyte. This is chosen for several reasons. Cardiomyocytes are highly important from a medical perspective, as cardiovascular disease remains the number one killer worldwide (Virani et al., 2020). As a result, developmental and disease-associated mechanisms controlling cardiomyocyte gene expression have been extensively studied for several decades (reviewed in Chang and Bruneau, 2012; Akerberg and Pu, 2019). Of particular relevance to this manuscript, cardiomyocyte 3D nuclear organization has been the focus of numerous recent investigations, an aspect my colleague and I recently reviewed (Bertero and Rosa-Garrido, 2020). Moreover, being a highly specialized, postmitotic cell type, the cardiomyocyte has a relatively stable 3D chromatin architecture that does not undergo the substantial rearrangements associated to the cell cycle (Nagano et al., 2013; Ramani et al., 2017), thus representing an ideal model to test the hypothesis outlined in this manuscript. On a practical level, the development of robust protocols to differentiate and mature cardiomyocytes from hESCs and induced pluripotent stem cells (iPSCs) has greatly simplified the study of cardiac biology, making this a widely accessible model (reviewed in Mummery et al., 2012; Protze et al., 2019; Karbassi et al., 2020).

### Cardiac Inter-Chromosomal Genome Organization

My colleagues and I found that differentiation of hESCs into cardiomyocytes leads to an almost two-fold increase of inter-chromosomal interactions, which represent ~50% of all interactions in hESC- and hiPSC-derived cardiomyocytes (Bertero et al., 2019a). These results were obtained using *in situ* DNase Hi-C, a genome-wide 3C approach that has less sequence bias and higher effective resolution than conventional, restriction enzyme-based Hi-C (Ramani et al., 2016). By detecting chromatin interactions within fixed and minimally altered nuclei, this method also minimizes the chances of detecting spurious inter-chromosomal interactions resulting from intermixing of chromatin (a problem characteristic of in-solution proximity ligation-based approaches, as discussed in the above section “Nuclear Subcompartments”; Nagano et al., 2015). Notably, the proportion of *trans* interactions in hESC-CMs remained consistently high over a broad range of fixation conditions (1–4% PFA), suggesting that *trans* contacts were not inflated by suboptimal capture of the native chromatin environment. A high proportion of *trans* interactions in cardiomyocytes was observed also in other *in situ* Hi-C experiments in cardiomyocytes from other hESC lines (~46%; Zhang et al., 2019), hiPSC lines (~60%; Bertero et al., 2019b), primary mouse cardiomyocytes (~39%; Rosa-Garrido et al., 2017), and fetal human hearts (~45%; Bertero et al., 2019a). Overall, cardiomyocytes appear to have marked inter-chromosomal genome architecture. This could be connected to their postmitotic state, which may facilitate the acquisition of a stable inter-chromosomal organization that is no longer periodically perturbed by the cell cycle. Whether the formation inter-chromosomal interactions follows changes in intra-chromosomal genome architecture or *vice versa* has not been yet clarified: establishing this will require sampling of differentiating cardiomyocytes at a much finer interval than what has been done so far.

Similarly to other cell types, cardiomyocytes present defined chromosome territories whereby small, gene-rich chromosomes are in closer proximity to each other than with large, gene-poor chromosomes (Bertero et al., 2019a,b). Accordingly, inter-chromosomal interactions are enriched for domains within the A compartment, and depleted for B compartment regions (Bertero et al., 2019a,b). Significant inter-chromosomal interactions are also markedly and specifically enriched for cardiac-specific genes (Chapski et al., 2019), suggesting that some *trans* interactions may involve the formation of functional chromatin subcompartments such as those described in the above section “Nuclear Subcompartments”.

One such example has already been presented in the above section “Splicing Factories”, namely the muscle-specific RBM20 “splicing factory” (Figure 2B), which is nucleated by its key target, the *TTN* pre-mRNA, and promotes alternative splicing of other targets on different chromosomes as they come in proximity with the *TTN* locus (Bertero et al., 2019a). Here it is worth adding that this structure is not just notable from a mechanistic standpoint, but may also play an important role in the regulation of cardiac function. Indeed, mutations in RBM20 are an established cause of dilated cardiomyopathy (DCM), a condition that leads to progressive impairment of cardiac output and ultimately to heart failure (Brauch et al., 2009). RBM20 mutations affect ~3% of DCM patients and lead to a particularly malignant form of the disease characterized also by conduction system disorders and/or life-threatening arrhythmias (in ~30 and ~45% of patients, respectively; Refaat et al., 2012; Haas et al., 2015; Kayvanpour et al., 2017; van den Hoogenhof et al., 2018). This is associated to a faster progression to heart failure and to the need of cardiac transplantation at a younger age than other DCM patients (Kayvanpour et al., 2017). Contractile dysfunction has been attributed primarily to splicing alterations in *TTN* (Guo et al., 2012; Maatz et al., 2014). Indeed, mutations in RBM20 lead to a longer, more compliant isoform of titin, a protein that plays a key role in maintaining cardiac stiffness by working like a molecular spring (Herman et al., 2012). The mechanism leading to electrical disturbances in the heart of RBM20 DCM patients is less understood, but may rely on other splicing alterations in ion-handling genes (Wyles et al., 2016a,b; van den Hoogenhof et al., 2018). In this context, a recent study by Schneider et al. (2020) found that a mutation of RBM20 in an arginine- and serine-rich hotspot leads to impaired nuclear localization and accumulation of mutant RBM20 in cytoplasmic RNP granules. Overall, it is tempting to speculate that maintenance of efficient RBM20 splicing factories is key to maintain cardiac homeostasis.

The cardiomyocyte nucleus is also characterized by numerous transcription factories. Analyses in mouse cardiomyocytes revealed that these respond dynamically to stress induced by humoral or mechanical stimuli (Karbassi et al., 2019). Moreover, this response is distinct in neonatal and adult cardiomyocytes: the former increase the number of actively elongating Pol II clusters, while the latter increase the size of existing ones. These transcription factory dynamics parallel the repositioning of cardiac genes that are up- or down-regulated in response to stress, which move closer to or away from the nuclear interior, respectively. Notably, promoter enrichment of Pol II binding is higher for loci closer to the interior. Moreover, integration of Hi-C data showed that genes upregulated after stress have a greater fraction of interaction with each other, and *vice versa* for downregulated genes (Rosa-Garrido et al., 2017; Karbassi et al., 2019). This collective evidence points towards the conclusion that the cardiomyocyte nucleus possesses transcription factories that are dynamically modulated in response to stress. Whether “zip coded transcription factories” (as defined in the above section “Transcription Factories”) are a common mechanism in this context remains, however, unclear.

It is important to mention that cardiomyocytes are well known as one of the few cell types that become polyploid in physiological conditions. Such process is a hallmark of postnatal maturation following withdrawal from the cell cycle (reviewed in Marchianò et al., 2019). This process is species-specific: while ~90% of rodent adult cardiomyocytes are tetraploid (4N) through bi-nucleation, with each nucleus being diploid (2N), ~75% of human adult cardiomyocytes remain mononucleated but grow in ploidy to 4N or, more rarely, 8N and even 16N. Stress can further augment human cardiomyocyte ploidy, particularly the proportion of 8N and 16N nuclei (Gilsbach et al., 2018). Therefore, it is conceivable that both developmental- and disease-associated ploidy changes may impose substantial changes on inter-chromosomal nuclear architecture. This aspect is, however, not straightforward to study using hESC- or hiPSC-CMs since these largely immature cells (resembling fetal or early postnatal developmental stages) are for the most part diploid (reviewed in Karbassi et al., 2020). Moreover, the aforementioned differences in rodent and human ploidy limits the predictive power of experiments in mouse models. Studies of ploidy in primary human cardiomyocytes have begun to emerge (Hesse et al., 2021), and may be the best approach to elucidate its impact on inter-chromosomal genome architecture.

### Cardiac *Trans*-Acting Expression Quantitative Trait Loci

As discussed in the above section “*Trans*-Acting Expression Quantitative Trait Loci”, the study of *trans* eQTLs requires a large sample size. Therefore, the identification of *trans* eQTLs in human cardiomyocytes has proved challenging due to the complexity associated to acquiring the necessary material from living individuals. Accordingly, the GTEx consortium only found a single *trans* eQTL from the analysis of 386 left ventricle samples (Aguet et al., 2020). On the other hand, the study of recombinant inbred rodent strains has provided valuable insights. For instance, analysis of 24 recombinant inbred mouse strains identified 1,357 *trans* eQTLs, as well as three clusters of *trans* eQTLs hotspots each regulating over 50 genes (Imholte et al., 2013). A similar analysis of a panel of 29 recombinant inbred rat strains led to the identification of 2,140 *trans* eQTLs for left ventricle tissue samples (Grieve et al., 2008). Notably, left ventricle *trans* eQTLs outnumbered by more than two-fold those found in the same study for fat, kidney, and adrenal tissue samples. The number of *trans* eQTL clusters was similarly highest for the left ventricle. Notably, left ventricle *trans* eQTL showed a larger degree of correlation in the expression of matched *trans* eGenes compared to that observed for *cis* eQTL-*cis* eGene pairs (3.4 vs. 0.6%, respectively). Such correlation was even more remarkable for left ventricle *trans* eQTL gene clusters, reaching 77.2%. Therefore, cardiomyocyte gene expression seems to be particularly affected by genetic variation in *trans*, providing further evidence for the possible role of inter-chromosomal genome organization in this cell type.

## A Bidirectional Link Between RNA Biogenesis And Nuclear Regulations In *Trans*?

Having introduced the key relevant observations in the previous sections, here I present and elaborate on the central thesis of this manuscript, namely that RNA biogenesis and regulation of inter-chromosomal genome organization are closely linked in a bidirectional fashion. As introduced earlier, this is by no means a novel idea *per se.* In fact, the contribution of transcription factories to 3D genome topology has been hypothesized at least a decade ago (Sexton et al., 2007; Cook, 2010). This concept has been extended by recent studies that coined terms such as “nuclear speckle hubs” or “regulatory communities” to describe emerging inter-chromosomal structures in various models (Dai et al., 2016; Quinodoz et al., 2018). In this context, my goal here is three-fold: (1) to generalize the “transcription factory hypothesis” of Cook, Pombo, Fraser, and others by also incorporating other RNA biogenesis steps beyond transcription; (2) to provide a partially distinct mechanistic framework; (3) and to outline the resulting predictions, to be used to test the overall hypothesis.

### A Unifying Hypothesis for RNA Factories and *Trans*-Interacting Domains

This hypothesis can be articulated in three central statements:

- All key steps involved in RNA biogenesis have the *potential* to contribute to inter-chromosomal genome architecture by leading to the formation of specialized RNA factories.
- RNA factories are sites where co-regulated nucleic acids, DNA and/or RNA, are found in close proximity to each other and to highly concentrated clusters of the factors that are key to their regulation.
- Engagements of nucleic acids with an RNA factory increase the fidelity and efficiency of their regulation.

Despite the evidence presented in the previous sections focused on mRNA biogenesis, the hypothesis described here may be generalizable also to other types of non-coding RNAs (such as rRNA in the nucleolus) since some of the underlying mechanisms (described in the next subsection) likely translate to the specific regulations involved in the biogenesis of various classes of RNAs. Moreover, while this hypothesis is articulated specifically for inter-chromosomal regulations, it likely also extends for intra-chromosomal ones. Indeed, there is abundant evidence that *cis* genome architecture involves clustering of co-regulated genes (Schoenfelder et al., 2010; de Wit et al., 2013; Delaneau et al., 2019).

With regards to the first statement, relevant RNA biogenesis steps include the regulation of transcription, as exemplified by the existence of specific regulatory inter-chromosomal interactions (sections “*Trans*-Acting Regulatory Regions” and “Polycomb Domains”) and of “zip coded” transcription factories (section “Transcription Factories”), but are not limited to this. Further regulation can be achieved by pre-mRNA processing steps in both specialized nuclear speckles (section “Nuclear Speckles”) and tissue-specific splicing factories (section “Splicing Factories”). Moreover, I anticipate that future studies will reveal how other aspects of RNA biogenesis, such as RNA post-transcriptional modifications, are similarly involved in regulating inter-chromosomal architecture.

With reference to the second statement, relevant regulatory factors include transcription factors (many examples; section “Transcription Factories”), chromatin modifiers (i.e., PRC2), genome organizers (i.e., CTCF), the transcriptional machinery (i.e., Pol II and its cofactors), splicing regulators (i.e., RBM20), and other protein factors involved in various steps of RNA biogenesis. RNA species with a structural and/or enzymatic role are also likely involved, though this will need to be determined by further studies.

While the term “RNA factory” describes the overall subnuclear structure, I propose that the genomic loci involved could be referred to as a “*trans*-interacting (chromatin) domain” (TID). This acronym purposely reminisces TAD in order to stress the conceptual similarity between these structural features of the genome. Similarly to TADs, TIDs are statistical constructs that can be observed only at a population level (Rowley and Corces, 2018; Beagan and Phillips-Cremins, 2020). In individual cells, interactions between loci within a given TAD or TID are necessarily limited by steric hindrance and are transient. Thus, both of these terms do not aim to define a stable structure, yet they describe a set of loci that are more likely to interact with each other than with different loci outside of the domain but within the same genomic context. Such definition is applied in *cis* for TADs, and in *trans* for TIDs. Notably, however, TIDs and TADs may partially intersect in that multiple *cis*-interacting loci within a TAD may contribute to a TID by engaging in inter-chromosomal interactions with the same set of *trans* loci. On average, the interaction probability for loci within a TAD is expected to be higher than that for loci within a TID, as *cis* interactions are generally favored. Importantly, beyond a certain genomic distance (10–50Mb, depending on the cell cycle stage), the probability of intra- and inter-chromosomal interactions becomes similar. Therefore, very long-range intra-chromosomal interactions may be effectively considered analogous to inter-chromosomal ones when computing and studying TIDs.

### Mechanisms Leading to the Formation of RNA Factories

**Figure 4:**
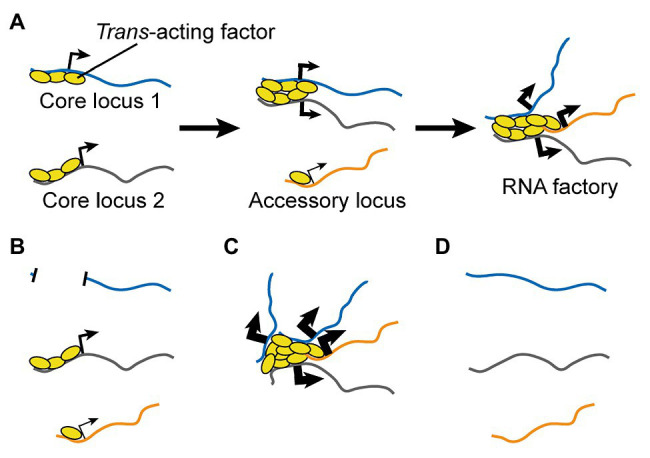
A Testable Model for the Formation of RNA Factories. **(A)** Multiple copies of *trans*-acting regulatory factors bind onto core nucleic acids, aggregate to form new clusters and/or enrich pre-existing ones, and recruit accessory co-regulated nucleic acids. RNA factories promote the efficacy and accuracy of RNA biogenesis processes (thicker black arrows). **(B-D)** The model can be tested by depleting **(B)** or adding **(C)** core binding sites for *trans*-acting regulatory factors, or by depleting the *trans*-acting regulatory factors. Loss-of-function perturbations are expected to impair inter-chromosomal interactions **(B,D)** and RNA biogenesis mechanisms **(B)**, while a gain-of-function experiment should have opposite effects (very thick arrows, **C**).

I propose a three-step process (Figure 4A):

- *Trans*-acting regulatory factors bind onto core co-regulated nucleic acids.
- Regulatory factors from multiple nucleic acids aggregate to form new clusters and/or enrich pre-existing ones.
- Accessory nucleic acids are brought into proximity the cluster until a dynamic equilibrium is reached.

Step one involves different factors for distinct classes of RNA factories. For transcription factories, *trans*-acting regulators include transcription factors, epigenetic remodelers, genome organizing proteins, and Pol II and its cofactors, which engage with DNA either directly or through its associated histones. For splicing factories, the key *trans*-acting factors, namely splicing regulators, require prior initiation of transcription to generate the target RNA molecules. In both cases, multiple binding sites are found on either the same or multiple molecules (in the case of RNAs). The model stipulates that one or few nucleic acids with the highest density of binding sites function as “core” element(s), dictating the local accumulation of the regulators.

Step two is the result of either active mechanisms (i.e., cytoskeletal dynamics) and/or Brownian movements of nucleic acids extruding from the core of chromosomal territories. Changes in the local chromatin environment may facilitate this process and increase intermixing in the inter-chromosomal space. Stabilization of core inter-chromosomal interactions involve either high-affinity interactions between factors that can form dimers and/or higher-order multimers (i.e., CTCF and many transcription factors), as well as weaker interactions such as those involving low-complexity regions characteristic of proteins that can undergo liquid-liquid phase separation (Patel et al., 2015). In some cases, regulators may join larger clusters that are pre-existing independently of nucleic acids.

In step three, large and growing clusters of regulators stabilize additional inter-chromosomal interactions with co-regulated nucleic acids characterized by lower binding density for the regulators. These “accessory” DNA or RNA molecules are those that most strongly benefit from interfacing with the RNA factory, which augments the efficiency of their regulation. As the RNA factory grows in size, it eventually hits steric and thermodynamic limitations that stabilize its further growth to a state of dynamic equilibrium.

Throughout the process, regulators can be either general and housekeeping, binding to nucleic acids with low degree of specificity or no specificity at all (i.e., CTCF and Pol II), or highly specific and responsive to cell states (i.e., tissue-specific transcription and splicing regulators). The resulting RNA factory is more or less cell state-specific depending on the relative balance of these two classes. “Zip coded” transcription and/or splicing factories result from highly specific regulators enriching for their target nucleic acids.

### Experimental Testing Strategies and Predicted Outcomes

Besides descriptive investigations, at least three types of perturbations can be used to test the model (Figures 4B-D):

- Depletion of core nucleic acids.
- Addition of clustered binding sites for *trans*-acting regulatory factors.
- Depletion of *trans*-acting regulatory factors.

The first experiment involves reducing or completely preventing the binding of *trans*-acting regulatory factor(s) to one or more of the core co-regulated nucleic acids. For DNA loci, this can be achieved by mutations or larger deletions. For RNAs, this can be achieved by mutations of the originating genes to prevent their transcription, or through epigenetic silencing. For both DNA and RNA perturbations, an important control experiment is to reduce the protein levels of the gene(s) of interest by a similar magnitude as the test perturbation, but without affecting the abundance of binding sites for the *trans*-acting regulatory factors. This is key to exclude that any observed effect is not simply due to impairment of intermediate protein-mediated regulations. For instance, one can generate knockout and/or hypomorph mutations that do not alter the DNA sequence in the regions of interest and/or do not alter RNA levels. The predicted outcomes of this perturbation are: (1) impaired clustering of the *trans*-acting regulators; (2) impaired inter-chromosomal interactions between loci in the TID; and (3) decreased efficiency in the regulation for accessory nucleic acids (Figure 4B).

The second experiment has the opposite goal, namely to increase the clustered binding of the *trans*-acting regulatory factor(s). For instance, one can express transgenic copies of a core nucleic acid or an artificial sequence containing arrayed binding sites. In the former case, the transgene can be mutated to prevent protein translation and bypass the need to control for this aspect. An RNA can also be upregulated through epigenetic mechanisms. The predicted outcomes of this perturbation are opposite to those outlined above (Figure 4C). Of note, this experiment can be also performed in combination to the previous perturbation in order to rescue the function of the RNA factory, so as to formally prove the sequence(s) that are necessary and sufficient for its formation and activity.

The third experiment is a simple loss-of-function perturbation for the *trans*-acting regulatory factor. In this context, the only testable aspect of the model is the expectation that this perturbation should reduce inter-chromosomal interactions for loci involved in the TID (Figure 4D). Indeed, loss-of-function of the regulatory factor is expected to reduce or abolish both its clustering and the regulation of its target nucleic acids independently of its function in an RNA factory.

## Testing the Hypothesis: Challenges and Ideas

To the best of my knowledge, only a handful of published studies on RNA factories have performed functional experiments along the lines of those proposed in the previous subsection. Examples include the aforementioned analyses of the TNFα-responsive transcription factory (section “Transcription Factories”; Fanucchi et al., 2013), and of the RBM20 splicing factory (section “Splicing Factories”; Bertero et al., 2019a). While the results of such investigations have been consistent with the expectations outlined above, much more work will be needed to thoroughly test the model proposed in the previous section and to identify its limitation. Moreover, the pervasiveness of this mechanism in the regulation of gene expression and inter-chromosomal genome organization will have to be established. Here I outline some of the key challenges towards meeting these two goals, and I propose some potential solutions.

### Identification of RNA Factories

Perhaps the most important roadblock in the field is the difficulty in predicting which inter-chromosomal interactions are functionally related to specific regulations of RNA biogenesis and not just background noise. Overcoming this issue will likely require refinements in both the experimental and analytical pipelines.

#### Methodological Consideration

From a methodological perspective, it is now clear that in-solution Hi-C is too noisy and therefore inadequate to probe for inter-chromosomal interactions (Nagano et al., 2015). Alternatives that rely on in-nucleus or substrate-tethered proximity ligation are therefore preferable (Kalhor et al., 2012; Nagano et al., 2015; Dai et al., 2016). An open limitation remains the lack of approaches that specifically detect *trans* chromatin interactions: since these can account for as low as 10% of the total ligation fragments and are spread over a large 2D genomic space, the costs to achieve a reasonable sequencing depth can be prohibitive. This is an even larger concern for single-cell Hi-C approaches, which result in data that is sometimes so sparse that even the analysis of *cis* interactions becomes a challenge (Nagano et al., 2013; Ramani et al., 2017). The development of strategies to enrich for inter-chromosomal proximity ligation fragments, for instance using capture probe sets for different chromosomes, may be a worthy pursuit. Along these same lines, it may be valuable to enrich for chromatin regions interacting with specific factors thought to be implicated in RNA factories, for instance using chromatin interaction analysis by paired-end tag sequencing (ChIA-PET; Fullwood et al., 2009) or proximity ligation-assisted ChIP-seq (PLAC-seq; Fang et al., 2016). Importantly, direct probing of nuclear RNA-RNA interactions in the context of RNA factories may be an important complementary approach, which may be enabled by recent methodological advances (reviewed in Kudla et al., 2020).

Another important technical consideration is the inability of Hi-C to reliably detect inter-chromosomal architecture when loci are not in very close and stable proximity, such as it may be the case for many RNA factories. Alternative ligation-free methods such as SPRITE, GAM, and TSA-seq have begun to elucidate inter-chromosomal architecture at varying spatial resolution (Beagrie et al., 2017; Chen et al., 2018; Quinodoz et al., 2018). Extension of these and similar approaches to also detect nuclear RNA-RNA interactions will be an important achievement. The application of emerging imaging approaches that allow simultaneous and/or sequential labeling of tens of loci will be key to validating the existence of RNA factories (Bintu et al., 2018; Mateo et al., 2019). Moreover, the recent development of high-throughput DNA FISH approaches gives hope that such methods may soon allow the necessary scalability required for discovery-type studies (Shachar et al., 2015; Finn et al., 2019).

#### Analytical Considerations

The identification of significant inter-chromosomal interactions must rely on statistical approaches that are specifically designed for this task. Indeed, approaches originally devised for the analysis of *cis* interactions control for biases that are not necessarily applicable to *trans* ones (for instance by correcting for the linear genomic distance between the interacting loci). Unfortunately, most of the analytical tools for 3C-type data developed so far have focused only on intra-chromosomal interactions (reviewed in Lin et al., 2019). The most recent implementation of FitHiC is one of the few publicly available tools that allows the user to interrogate bulk Hi-C data for both *cis* and *trans* interactions (Ay et al., 2014; Kaul et al., 2020). This approach makes minimal assumptions about genome size or structure, and generates an empirical null model to estimate the significance of interactions. While robust and flexible, this strategy does not account for known biases of *trans* interactions in mammalian systems, such as their enrichment for loci in the A compartment and their different distribution across small and large chromosomes. A similar limitation applies to existing network-based strategies to study inter-chromosomal interactions both from bulk Hi-C (Kaufmann et al., 2015) and single-cell Hi-C data (Bulathsinghalage and Liu, 2020). Statistical frameworks that explicitly model chromosome territorialization and chromatin (sub)compartmentalization may lead to more streamlined identification of inter-chromosomal interactions arising from metastable RNA factories, which are expected to “stand out” from other *trans* interactions resulting from the random intermixing of neighboring chromatin domains.

Another promising venue is the modeling and study of 3D genome structure populations, which as discussed in the section “Nuclear Subcompartments” has already provided important insights in inter-chromosomal genome architecture (Kalhor et al., 2012; Dai et al., 2016). Nevertheless, these models are currently limited in their spatial resolution due to the computational complexity involved in finely modeling large mammalian genomes. Besides presenting its own important statistical challenges, analysis of imaging-based chromatin structure data is also currently not easily integrated with sequencing-based data modalities. This remains is one of the most important areas for further analytical development in the wider 3D chromatin organization field.

### Identification of *Trans*-Acting Regulators in RNA Factories

A second key challenge towards clarifying the role of RNA factories is the identification of the *trans*-acting factors that contribute to their formation and/or function. For transcription factories, the enrichment of DNA binding motifs within the regulatory regions proximal to the involved loci may provide testable hypotheses as to the transcriptional regulators involved. RNA binding motifs enrichment on pre-mRNAs may similarly help in the context of RNA splicing factories. Further evidence could be gathered by integration of data modalities directly probing for DNA and/or RNA binding of candidate factors. However, these analyses are limited by the availability of well-annotated binding motifs and/or of experimentally determined binding profiles for the cell state of interest.

Robust methods to unbiasedly identify factors that interact with specific nucleic acids are direly needed. Biochemical purification approaches relying on direct capture of nucleic acids have been developed, but so far showed limited sensitivity and specificity (reviewed in Machyna and Simon, 2018). A potential alternative is the application of *in situ* proximity labeling, for instance by relying on promiscuous biotinylating enzymes to covalently tag nearby proteins for subsequent streptavidin-based purification and identification through mass spectrometry. These methods have been successfully used to determine protein-protein interactions, and their implementation to study locus-specific regulations are also emerging (reviewed in Ummethum and Hamperl, 2020).

### Testing the Function of RNA Factories

Last but not least, the functional characterization of RNA factories is key to determining their physiological relevance. As elaborated in the section “Experimental Testing Strategies and Predicted Outcomes”, mechanistic studies may involve various means of perturbing RNA factories through loss- and/or gain-of-function. While these studies do not pose unsurmountable challenges due to recent advancements in genome editing technologies, they remain time-consuming. Characterization of putative RNA factories may be performed with more throughput by leveraging on functional screening strategies based on the combination of CRISPR/Cas9 perturbations and transcriptional phenotyping through single-cell RNA-sequencing (reviewed in Yao and Dai, 2018).

## Conclusion

In summary, emerging evidence suggests a functional bidirectional link between the regulation of RNA biogenesis and inter-chromosomal 3D genome architecture. In other words, the formation of RNA factories organized around TIDs may be not just an emergent property of gene regulation, but also a feature needed to maximize the fidelity and efficiency of such regulations. This is a testable hypothesis based on a clear mechanistic model: the clustered binding of *trans*-acting regulators onto core nucleic acid targets, leading to their aggregation and to the subsequent recruitment of accessory co-regulated nucleic acids. Future studies will prove, disprove, or modify this model. It will also be key to test the pervasiveness of such regulation to determine whether it involves only a few specific nucleic acids or it is a general property controlling many loci.

Many key open questions remain in this area. First off, what is the biochemical and/or biophysical mechanism that explains the co-localization of certain nucleic acids from different chromosomes? Besides transcription factors (Dai et al., 2016, 2018), several DNA binding proteins may be involved, including CTCF (Ling et al., 2006; Botta et al., 2010), Condensin II (Rowley et al., 2019), ARID1A (Wu et al., 2019), and the Pol II machinery (Nagashima et al., 2019). Regulatory RNAs may also play a key role. Such factors may form higher-order clusters *via* a combination of specific and non-specific interactions, possibly leading to liquid-liquid phase separation (reviewed in Banani et al., 2017; Langdon and Gladfelter, 2018). Clustering may happen after progressive concentration onto specific nucleic acids with high avidity for the factors. Alternatively, pre-existing clusters may “trap” target nucleic acids as they come into contact through Brownian motions. Active repositioning of loci through the nuclear cytoskeleton may be also involved (Hu et al., 2008). Likely, multiple mechanisms contribute to different extents in various cases.

Is dysfunction of RNA factories and/or TIDs involved in the pathogenesis of human disease? Cancer has been linked to alteration of chromosome territories (Barutcu et al., 2015) and of specific inter-chromosomal interactions (Patel et al., 2014; Du et al., 2015). Disruption of inter-chromosomal chromatin interactions has been observed in a human model of 15q duplication syndrome, an aneuploidy linked to 1–3% of autism cases (Meguro-Horike et al., 2011). Viral genomes can also engage in specific inter-chromosomal associations, as shown for Epstein-Barr Virus (EBV; Moquin et al., 2017) and Kaposi’s Sarcoma-Associated Herpesvirus (KSHV; Kang et al., 2011). Whether any of these or other diseases involve the dysregulation of RNA biogenesis factories remains to be tested. Notably, disruption of RBM20 splicing factories may play a key role in the pathogenesis of DCM (Schneider et al., 2020), though whether RBM20 mutations disrupt 3D chromatin organization is unclear.

Moving beyond RNA biogenesis, is proximity between co-regulated nucleic acids a general phenomenon in biology? It is tempting to speculate that other key biochemical reactions such as DNA repair and replication may also benefit from the increased efficiency and fidelity led by local accumulation of relevant regulators. Overall, increasing our spatial awareness of nuclear regulations may prove a worthy and impactful pursuit.

## Data Availability Statement

The original contributions presented in the study are included in the article/supplementary material, further inquiries can be directed to the corresponding author.

## Author Contributions

The author confirms being the sole contributor of this work and has approved it for publication.

### Conflict of Interest

The author declares that the research was conducted in the absence of any commercial or financial relationships that could be construed as a potential conflict of interest.
